# Dome-like behaviour at Mt. Etna: The case of the 28 December 2014 South East Crater paroxysm

**DOI:** 10.1038/s41598-017-05318-9

**Published:** 2017-07-13

**Authors:** C. Ferlito, V. Bruno, G. Salerno, T. Caltabiano, D. Scandura, M. Mattia, M. Coltorti

**Affiliations:** 1 Università degli Studi di Catania, Dipartimento di Scienze Biologiche, Geologiche ed Ambientali, Corso Italia 57, Catania, 95129 Italy; 2 Istituto Nazionale di Geofisica e Vulcanologia, Sezione di Catania, Osservatorio Etneo, Piazza Roma 2, Catania, 95125 Italy; 3 Università degli Studi di Ferrara, Dipartimento di Fisica e Scienze della Terra, Via Saragat 1, Ferrara, 44122 Italy

## Abstract

On the 28 December 2014, a violent and short paroxysmal eruption occurred at the South East Crater (SEC) of Mount Etna that led to the formation of huge niches on the SW and NE flanks of the SEC edifice from which a volume of ~3 × 10^6^ m^3^ of lava was erupted. Two basaltic lava flows discharged at a rate of ~370 m^3^/s, reaching a maximum distance of ~5 km. The seismicity during the event was scarce and the eruption was not preceded by any notable ground deformation, which instead was dramatic during and immediately after the event. The SO_2_ flux associated with the eruption was relatively low and even decreased few days before. Observations suggest that the paroxysm was not related to the ascent of volatile-rich fresh magma from a deep reservoir (dyke intrusion), but instead to a collapse of a portion of SEC, similar to what happens on exogenous andesitic domes. The sudden and fast discharge eventually triggered a depressurization in the shallow volcano plumbing system that drew up fresh magma from depth. Integration of data and observations has allowed to formulate a novel interpretation of mechanism leading volcanic activity at Mt. Etna and on basaltic volcanoes worldwide.

## Introduction

Among the volcanological community, a well-defined distinction between closed system and open-conduit volcanoes has long been accepted^1, 2^. It is common understanding that volcanoes can behave either as closed systems with batches of magma residing at crustal depth and able, all of a sudden, to break the volcanic edifice and cause violent explosive eruptions^3–5^ or open conduit volcanoes that are persistently filled with magma degassing from the summit vents^3, 6–8^. Degassing in basaltic magma is relatively easy because of the low viscosity of the melt and therefore vesicle coalescence may create bubbles large enough to decouple from the melt and reach the surface^9, 10^. On the contrary, in closed-systems typically associated with evolved magmas, such as andesite and rhyolite, high viscosity prevents efficient bubble coalescence and gas transport mainly occurs via permeable flow through fractures or connected vesicles^6, 11–13^. In such systems, the low permeability can often produce sealing of the pathways thereby preventing gas escape and causing pressure build-up^14, 15^.

**Figure 1:**
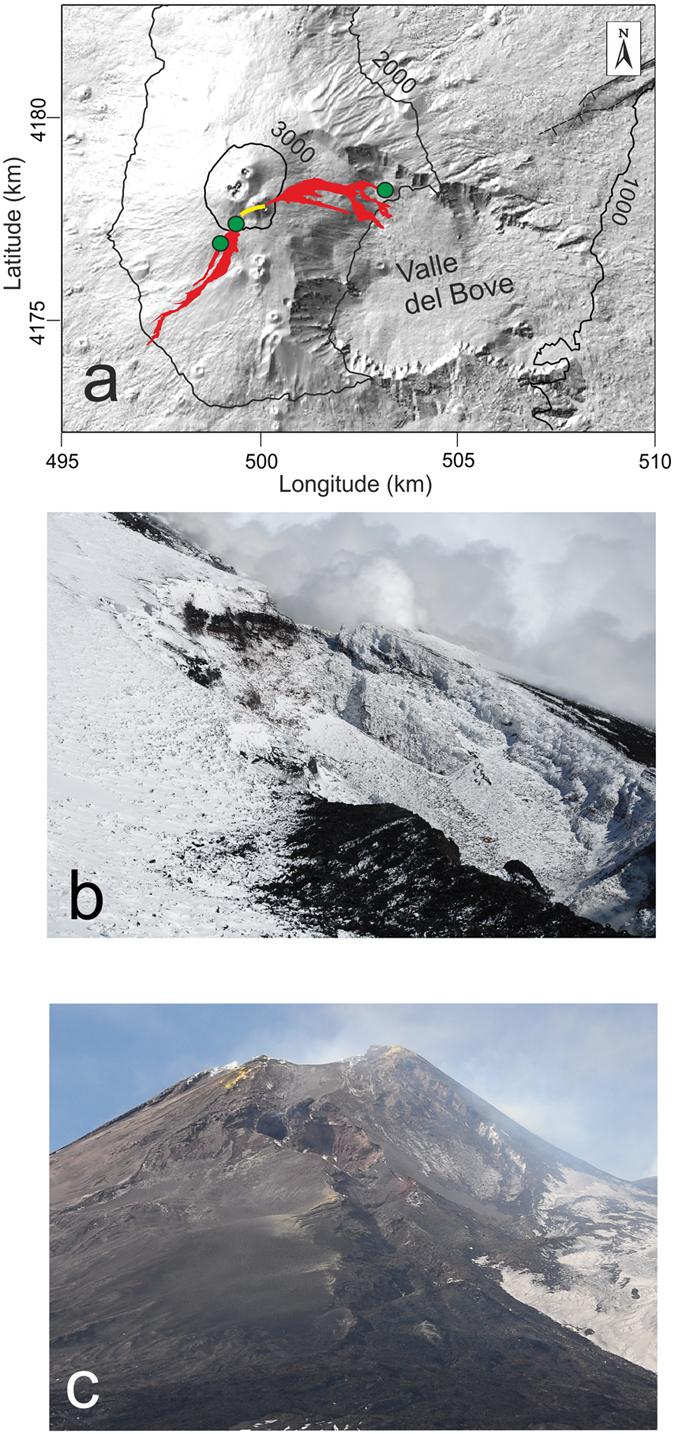
(**a**) Map of the uppermost portion of Mt. Etna volcano and the lava flow field of the 28 December eruption (red); yellow track represents the eruptive fracture; green circles are the rock sampling spots. (**b**) the large “avalanche” scallop representing the tip of the south-western branch of the eruptive fracture (~3050 m a.s.l.) (for dimensions see Supplementary Fig. S2); (**c**) large “avalanche” scallop representing the tip of the north-eastern branch of the eruptive fracture (~3050 m a.s.l.). Figure (**a**) was generated using CorelDRAW graphic suite software (http://www.corel.com/it/). The topography is based on a DEM held by INGV-OE, Cartography Lab (http://geodb.ct.ingv.it:8088/geonetwork/srv/ita/main.home). Photos taken by C. Ferlito.

**Figure 2:**
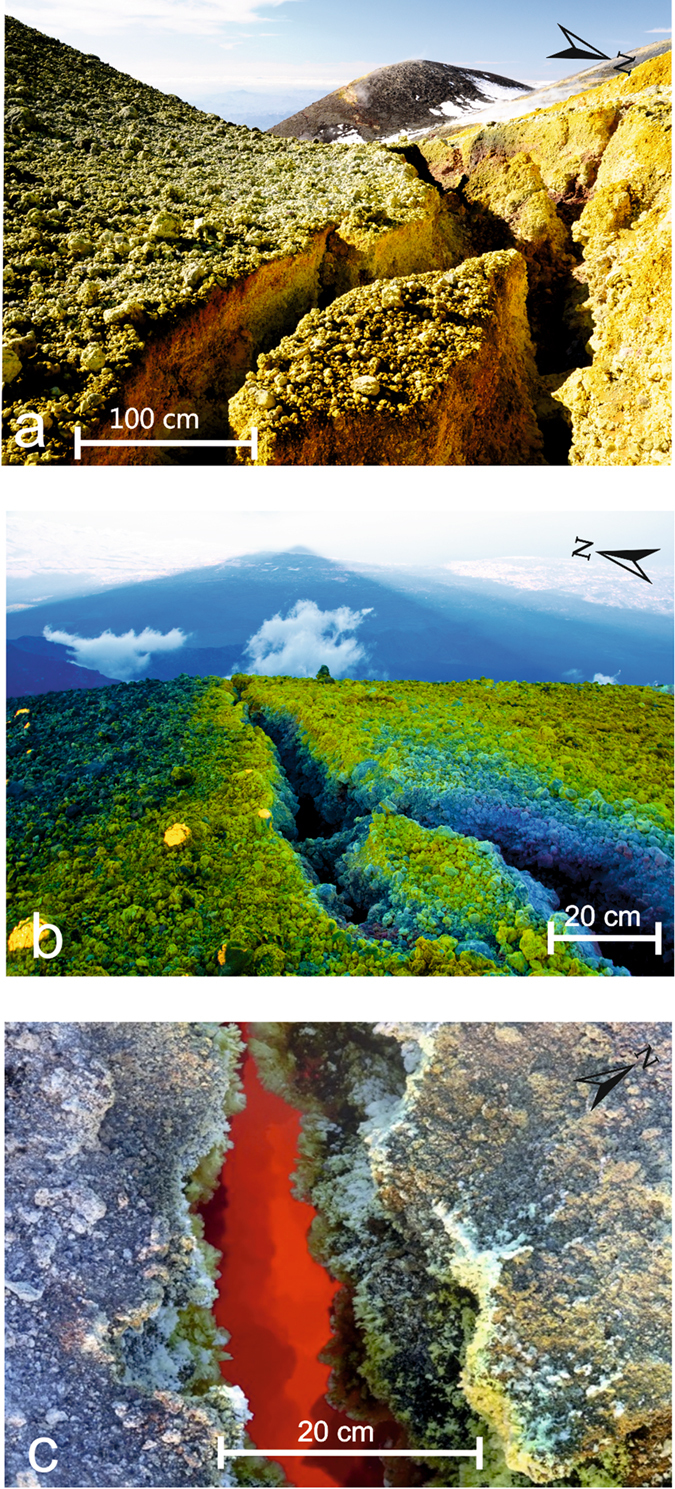
(**a**) Photo of the fractures field on the northern ridge of the SEC, the average width of the fractures was about 1 m, totally fumarolized and covered with sulphate sublimates that produce a bright yellow colour (photo taken on 1 November 2014); (**b**) photo of the fractures on the eastern ridge of the SEC summit; (**c**) incandescent fracture on the eastern ridge of the SEC (photo taken on 15 November 2014). Photos taken by C. Ferlito.

Mount Etna is a basaltic open conduit volcano whose terminal conduit is formed of four summit vents: the Voragine (VOR), the oldest and main crater; the Bocca Nuova (BN), located on the same cone of the VOR and by two sub-terminal cones the North-East Crater (NEC) and the South-East Crater (SEC) (Fig. 1a, Supplementary Video V1). In recent years, most of the open conduit summit eruptions have occurred at SEC and consisted of tens of “paroxysms” (44 since 2011), which are short-lived, high-energy explosive events, often culminating in lava fountaining, characterized by the emission of fresh gas-rich basaltic magma (e.g. Behncke *et al*.^16^; Patanè *et al*.^17^, De Beni *et al*.^18^). Since 2007, this significant activity at the SEC has shifted its main emission point of few hundred meters to the east of the SEC summit. This has been interpreted by some authors as the development of an independent eruptive axis and named as New South East Crater (NSEC, e.g. Behncke *et al*.^19^; Acocella *et al*.^20^). In this work, we will study the paroxysm occurred at the SEC the 28 December 2014. This eruption was previously studied by Bonforte & Guglielmino^21^ and Gambino *et al*.^22^, who classically interpreted the event as fed by a magmatic dyke. However, the event was marked by an extremely high discharge rate (~370 m^3^/s) and the two eruptive vents, at the base of SEC (Fig. 1a), were located at the bottom of large avalanche scallops (Fig. 1b,c; Supplementary Figs. S1 and S2); months before the paroxysm on the summit of the SEC we detected a complex network of incandescent fractures (Fig. 2; Supplementary Fig. S3). Moreover, the ground deformations measured by Continuous GPS (CGPS) were very sizeable during and after the event (not immediately before); seismic events, often characterizing the opening of eruptive fractures^23^ occurred only during the event (not before)^22^; the gas release was considerable days after the event (not during or before). The integrated data from field observations, geophysics, gas geochemistry and rock petrochemistry, allow inferring that the eruptive mechanism of the event of 28 December 2014, differs from open conduit paroxysms and dyke-fed eruptions but instead points to the behaviour of an exogenous dome. Such a behaviour, anomalous for basaltic systems, is physically detailed here for the very first time on Mt. Etna.

### Volcano-tectonic setting of Mt Etna and the eruptive event of 28 December 2014

The SEC originated as a pit crater during the 1971 eruption. It has been highly active since 1978^24, 25^ and has grown, over the last 10-12 years^16, 26^ to reach a height comparable to that of the Central Crater (3280 m a.s.l.). The last eruption at the SEC, before the event of 28 December 2014, occurred in August and consisted of strombolian activity lasting one week (from 8 to 15 August, 2014). It was accompanied by a lava flow issuing from the eastern flank of the SEC directed towards the Valle del Bove^18^. At the end of this activity, the summit of the SEC was affected by progressive fracturing, which became more intense in time and developed a network of large gas releasing fractures. Those fractures were coated by sulphur sublimates and showed incandescence months before the paroxysm (Fig. 2; Supplementary Fig. S3). The eruption of 28 December 2014 started, according to the sharp increase of volcanic tremor, at ~16:15 (all times are in GMT), when the volcano was entirely covered with clouds and direct observations could not be made. The paroxysm lasted more than two hours and its ending was revealed by a sharp decrease in volcanic tremor at 18:30. During the eruption, two distinct lava flows, for a total volume of ~3 million m^3^, as estimated by INGV-OE^26^, were emitted from the two tips of a fracture, both located at an elevation of about 3050 m a.s.l. (Fig. 1a). Considering the short time of the event, the discharge rate that can be inferred is ~370 m^3^/s; the two lava flows expanded for a distance of ~5 km from the SW tip and ~3.5 km from the NE tip, into the Valle del Bove. The thickness of the lava flows was around one meter close to the vents, increasing at the very distal front of the flows. The lava field was “aa” type, totally breached and clinkery, without any “pahoehohe” morphology and most important the lava flow was almost lacking the massive portion. The petrochemical features of the lava emitted do not differ from the high-K trachybasaltic lavas ordinarily produced by Etnean activity, except for a slightly higher Ca tenor and for the extremely low H_2_O content (see Tab 2). One peculiar feature of this paroxysm is the newly opened fracture, characterized by the alignment of huge niches similar to avalanche scallops; two of which broke apart the SW flank of the SEC (Fig. 1b; Supplementary Figs S1 and S2) and one broke the NE flank (Fig. 1c), for a total length of about 1200 m. Moreover, a network of minor fractures, cutting the erupted products, accompanied the main niches (Supplementary Fig. S3, Supplementary Video V1). A few days after the event (January 2, 2015), the emission of dense clouds of brown ash started from the SEC summit (Supplementary Fig. S7). On 5 January, a strombolian activity took place at the VOR, which had been inactive for years (Supplementary Fig. S5). This strombolian activity ended on 9 January.

## Results

### Deformation and modelling of volcanic sources

**Figure 3:**
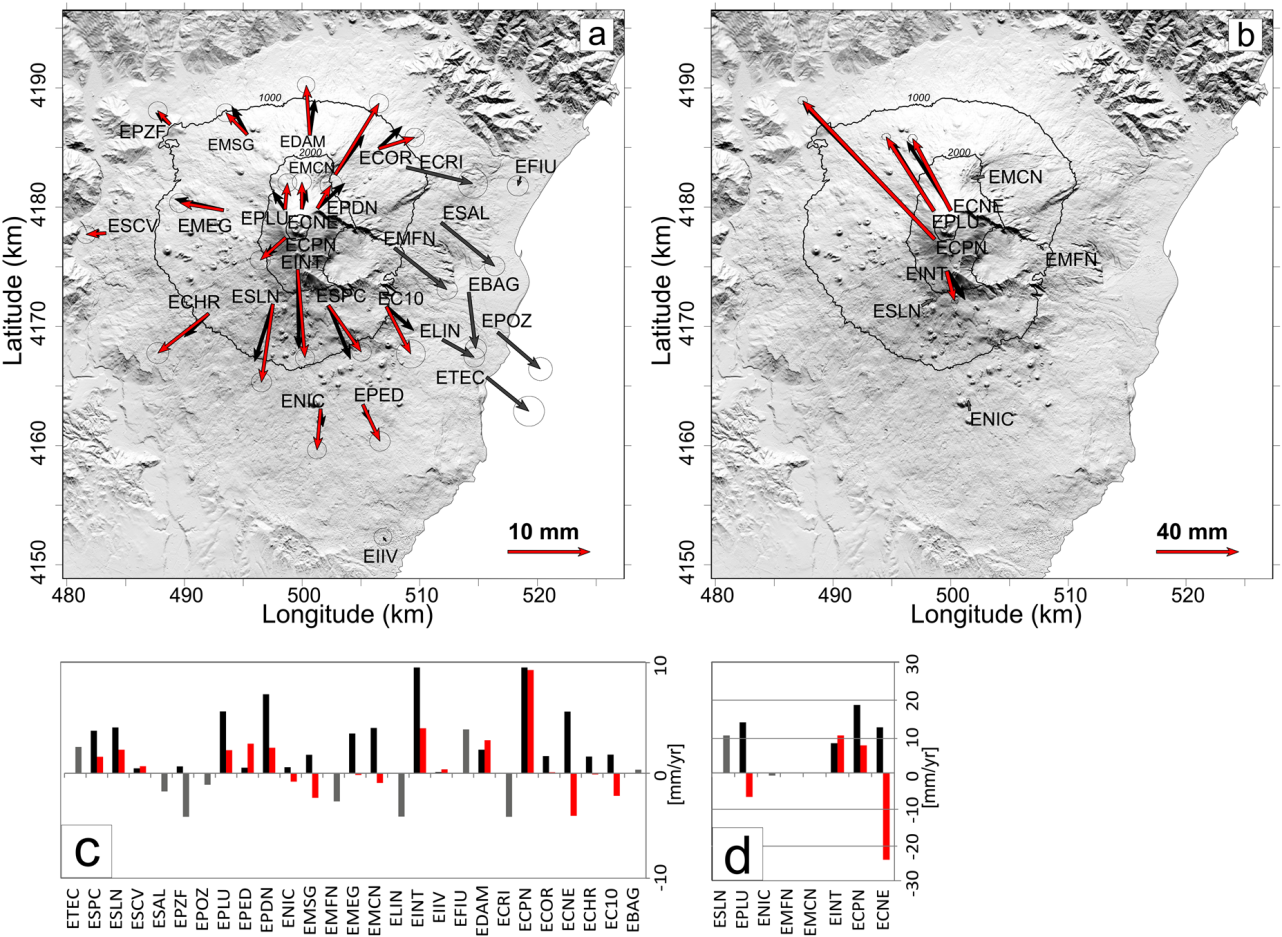
Recorded (red and grey arrows) and modelled (black arrows) horizontal CGPS displacements for the periods (**a**) 21 August - 25 December 2014, (**b**) 27–29 December 2014. Grey arrows were not considered in the modelling because the CGPS stations are on the eastern flank of Mt. Etna, characterized by a southeastward displacement not directly related to volcanic activity^61^; (**c**,**d**) the histograms show the recorded (red bars) and modelled (black bars) vertical displacements for each of the two phases respectively. This figure was created using CorelDRAW™ graphic suite software (http://www.corel.com/it/). The topography is based on a DEM held by INGV-OE, Cartography Lab (http://geodb.ct.ingv.it:8088/geonetwork/srv/ita/main.home).

CGPS network (Supplementary Fig. S5) data were used to: (i) explore and model the source of the ground deformation pattern preceding the paroxysm of 28 December 2014; (ii) explore and model the displacements following the paroxysm. The length increase (~15 mm since 21 August 2014) of the baseline between EMSG and ESLN CGPS stations, crossing the volcanic edifice from North to South indicates the inflation of the volcanic edifice (Supplementary Fig. S6a). This is also marked by the southward displacement of the EINT station (Supplementary Fig. S6b), located in the upper portion of the southern flank of the volcano, and by the summit station ECPN (Supplementary Fig. S6c). From 28 to 29 December 2014, the EMSG-ESLN baseline and the EINT and ECPN time series, showed a displacement due to the opening of the eruptive fractures (Supplementary Fig. S6). Supplementary Fig. S6c also shows the north-westward displacement of the ECPN station by ~102 mm. The displacement increased until the following day, with a variation in the North component of ~56 mm. Conversely, EINT station moved southwards by ~7 mm on 28 December and by ~4 mm the next day (Supplementary Fig. S6b). The ground deformation patterns in the period between 21 August–26 December and 27–29 December 2014 are shown in Fig. 3a,c and in Fig. 3b,d respectively. The CGPS displacements in the first period display a radial pattern (Fig. 3a), testifying the inflation of the plumbing system. Between 27 and 29 December, displacements were observed only in the summit area (Fig. 3b). In particular, EPLU and ECNE stations displaced toward NNW by ~47 and 44 mm respectively, ECPN moved NWward by ~102 mm and EINT, moved to the south by ~17 mm.

**Figure 4:**
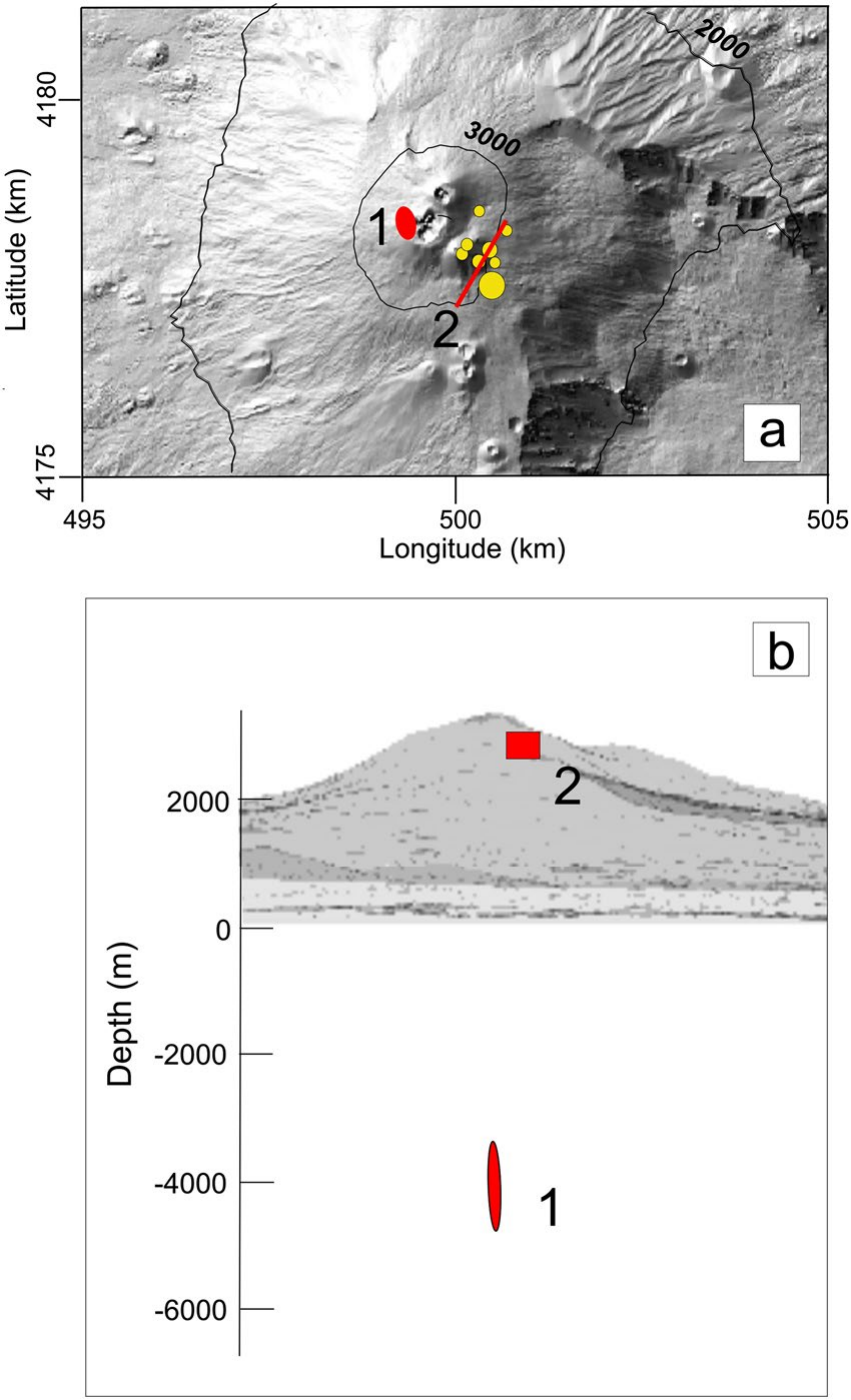
Surface (**a**) and vertical projection (**b**) of the analytical models of the deformation patterns. Inflating pressure source (1) for the first period (21 August - 25 December 2014) and tensile dislocation (2) related to the 28 December 2014 event. The yellow circles indicate seismic event locations (small circles: LP events taking place on 28 December at ~16:00–17:00; big circle: Ml = 3 event occurring on 28 December at 16:50). This figure was created using CorelDRAW™ graphic suite software (http://www.corel.com/it/). The topography is based on a DEM held by INGV-OE, Cartography Lab (http://geodb.ct.ingv.it:8088/geonetwork/srv/ita/main.home).

The deformation patterns of Fig. 3a,c,b and d were interpreted with analytical time-invariant models. We obtained spatial coordinates and geometrical parameters from data inversions and the uncertainties of each optimized source parameters have been evaluated using the Jack-knife re-sampling methodology^27^ (Table 1). For the first period, we modelled an inflating pressure source, associated with a volume of 9.6 × 10^6^ m^3^ (Table 1), at a depth of 4029 ± 654 m (b.s.l.) underneath the summit craters area (Fig. 4a,b). On 28 December the eruptive event occurred, right after which we observe a dramatic deformation of the summit area, lasting until 29 December (Supplementary Fig. S6b,c). We modelled the deformation source as tensile dislocation 795 ± 30 m long and 502 ± 17 m wide underneath the area just to the SE of the SEC, at a depth of 2672 ± 180 m (a.s.l.).

**Table 1:** Model parameters and related uncertainties: x_c_, y_c_ and z_c_ are the coordinates of the sources, *θ* is the azimuth measured counterclockwise from the positive y direction around the z axis, *ϕ* is the dipping angle measured clockwise from the positive y direction around the x axis, *a* is the major semiaxis, *b/a* is the ratio between the minor and major axes, P is the intensity of the pressure on the surface of the spheroid, ∆V is the changing volume calculated according to Tiampo *et al*.^62^ using a value of the effective shear modulus *µ* equal to 5 GPa.

Parameters	Phase 1	Phase 2
	*Source 1*	*Source 2*
x_c_ [m]	499763 ± 342	500131 ± 288
y_c_ [m]	4178210 ± 304	4177711 ± 683
z_c_ [m]	−4029 ± 654	2672 ± 1,8
*θ* [°]	10,4 ± 8,7	212 ± 34
*ϕ* [°]	103 ± 6	89 ± 3,7
*a* [m]	766 ± 142	
*b/a*	0,5 ± 0,13	
*l* [m]		795 ± 30
*w* [m]		502 ± 17
*Opening* [m]		1.85 ± 1.7
P [Pa]	1,5E + 09 ± 0.6 + 09	
ΔV [m^3^]	9,6E + 06

Our model differs from the one proposed in Gambino *et al*.^22^. Although most parameters are similar, our modelled source contains the area where seismic events occurred. But the most important difference lies in the interpretation of the role that these syn-post eruptive deformations played (see Discussion section).

### SO_2_ Flux

**Figure 5:**
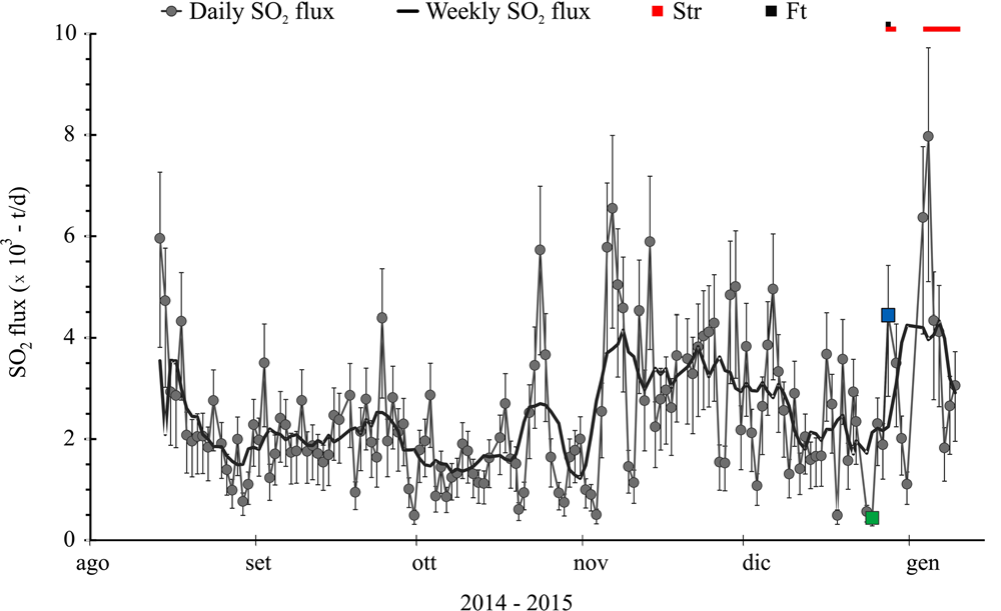
Daily (solid grey line) and weekly (black line) bulk SO_2_ flux between 14 August 2014 and 09 January 2015). Solid boxes at the top of the graph indicate the Strombolian (Str) and lava fountain (Ft) activity occurring throughout the investigated period. The solid green and blue squares in the daily SO_2_ flux are the minimum of the time series and the onset of the 28 December eruptive episode, respectively.

In order to better constrain the eruptive event of 28 December 2014, we have also examined the bulk SO_2_ flux released by the Etnean summit craters from the end of the previous eruptive event (15 August, 2014) to 09 January 2015 (Fig. 5). In the first 67 days (14 August-20 October), the daily flux remained stable at ~2000 tons per day (t/d). Since 21 October, the flux increased, leading to values up to 6500 t/d; this trend continued until the 7 December, when it gradually changed, decreasing to a mean value of 1800 t/d, down to the minimum of 440 t/d recorded on Christmas Day; this is the lowest flux measured in the studied period. In the three following days, the flux increased steadily to reach the value of 4500 t/d on the day of the eruption. It is worth mentioning that the SO_2_ flux recorded during the eruption was lower than the peaks recorded in November. Right after the eruption, the flux dropped to ~1100 t/d on 31 December, to increase again dramatically up to the 8000 t/d on 2 January 2015. This flux value is the maximum value of the time series and was measured in correspondence of the short but significant strombolian activity at the VOR, which lasted until 9 January.

## Discussion

Although the eruptive activity at Mt. Etna has prevalently been effusive, in the last few years the SEC has displayed a switch to short-lasting explosive eruptions. Since its formation as a pit crater in 1971, the SEC has undergone an articulated growth^18^, mainly due to its explosive-oriented eruptive style, which strongly increased since 2011^17, 19, 28^. Due to this intense activity in particular since 2013, its crater morphology has dramatically changed^18^, with the lack of a proper deep chasm replaced instead by a shallow and flat hollow constellated of fumaroles. The process behind this change has not been investigated yet; nevertheless, it must be considered that morphology on volcanoes is generally controlled by endogenous factors. As an example, a deep chasm within a crater is originated by the collapse, at the end of the eruption, of the magma filling the conduit. However, in order to be literally drawn back and leave a conduit empty, the magma needs to be fluid enough. It follows that the lack of a deep chasm at the SEC is likely due to the recurrent formation of viscous/rigid plugs of magma. A useful analogy can be found in Ferlito *et al*.^29^, who reported, during the 2001 eruption at the Laghetto parasitic cone, a sudden increase in viscosity and yield strength of the flowing lava, without any compositional variation. This behaviour was explained by invoking an in-conduit crystallization of microlites owing to magma undercooling in response of gas loss. Similar conditions could have occurred at the SEC, as evidenced by the large gas emissions associated with paroxysms of these last years. The 28 December 2014 eruption displayed features diverging from the classic basaltic unrest^17, 30, 31^, resembling instead those expected in a “dome-like” scenario. One of these features relates to ground deformations, which consisted in only a moderate inflation powered by a source located at about 5 km b.s.l., months prior to the onset of the eruption (Fig. 3a,c), and not associated to the ascent of fresh magma from depth. Interesting considerations can be made by looking at the borehole strainmeter data^32^, which revealed variations of the strain field with opposite polarity and diverse magnitude two hours before (13:50) and at the onset of the eruption (16:15), respectively. We interpreted these variations as: 1) an earlier positive strain (contraction), associated with the rapid vesiculation and pressurization of the plug of magma at the shallow level of the volcanic edifice; followed by 2) a negative strain (dilatation), associated with removal of the plug.

**Figure 6:**
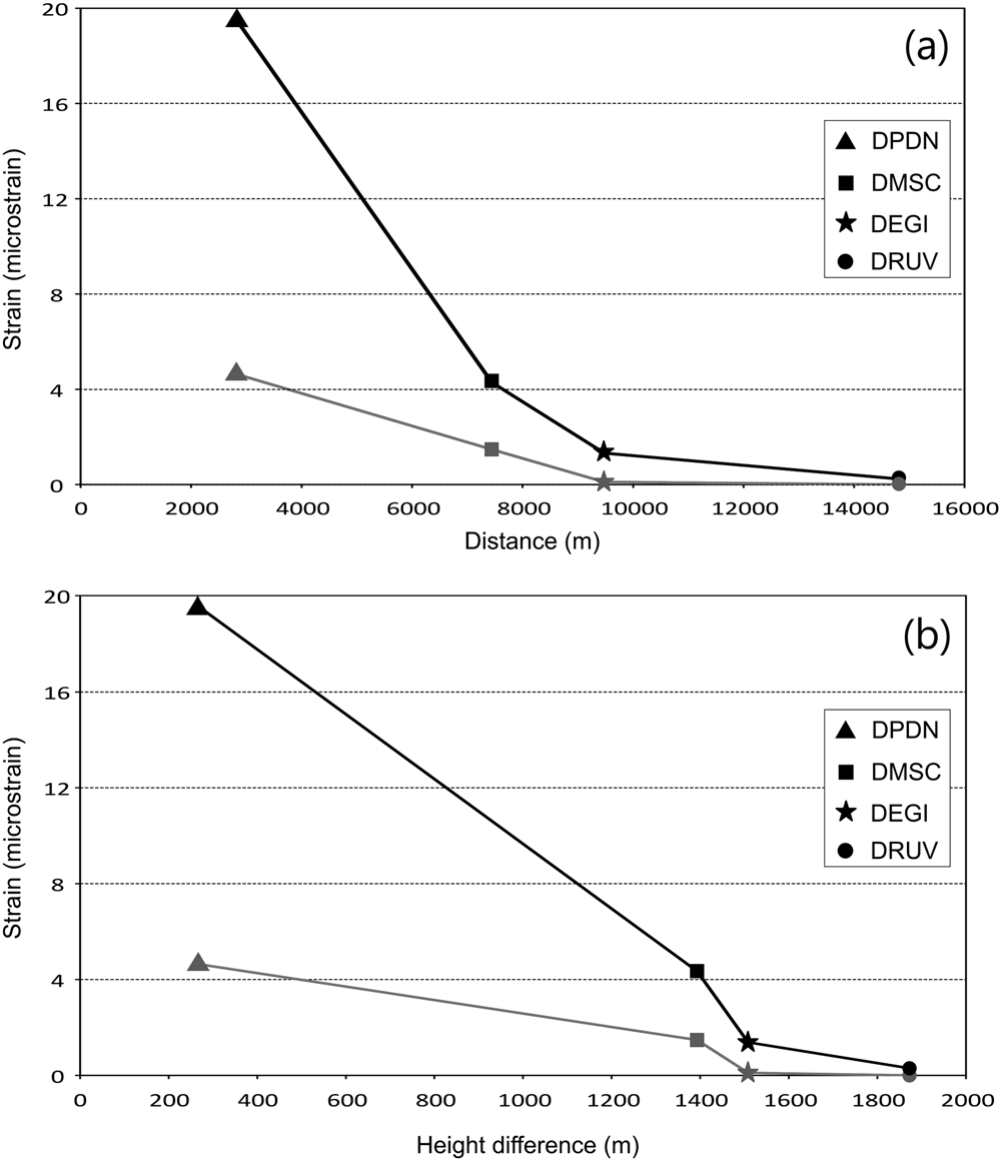
Strain vs. Distance (**a**) and vs. Altitude difference (**b**), between the SEC summit and the borehole dilatometers measuring stations (the data are inferred from figure 8 in Bonaccorso *et al*.^32^). Stations are located on the flanks of the volcanic edifice (DPDN, Pizzi Deneri −2800 m a.s.l.; DMSC, Monte Scavo −1740 m a.s.l.; DEGI, Monte Egitto −1600 m a.s.l.; DRUV, Monte Ruvolo −1400 m a.s.l.). The grey lines indicate the contractional (positive) strain during the couple of hours preceding the eruptive event. The black line represents the dilatational (negative) strain during the eruptive event. The two signals have different maximum intensity: the compressive signal is only ~4 microstrain, whereas the dilatational signal reaches ~20 microstrain.

Noteworthy is the magnitude of the strain at the four stations (Supplementary Fig. S5). Both positive (contraction) and negative (dilatation) strains show the highest values at the Pizzi Deneri station (DPDN), the closest to the SEC summit (about 2.5 km northwards). Values rapidly drop with increasing distance from the SEC at the Monte Scavo, Monte Egitto and Monte Ruvolo (DMSC, DEGI and DRUV, respectively) stations (Fig. 6). This behaviour testifies to a rapid decay of the measured volumetric strain, which can only be originated by a very shallow source, likely within the uppermost 1300 m of the Etnean edifice. SO_2_ flux emission also showed peculiar features, since mid-October 2014, bulk SO_2_ emission increased, to drop to a minimum value of 440 t/d three days before the eruption. The day of the eruption the flux increased up to 4500 t/d, which is lower than observed in November (Fig. 4). This syn-eruptive flux is anomalously low if compared with what was measured during SEC paroxysms (~15000 t/d^17, 33^).

Such geophysical and geochemical features suggest that the 28 December eruption was not associated with fresh volatile-rich magma migrating from depth but, conversely, was related to the removal of a plug of semisolid and degassed magma placed in the uppermost portion of the Etnean plumbing system, probably after the previous eruptive event of August 2014^34, 35^. Moreover, it is reasonable to suppose that, due to the typically low thermal conductivity of basalt, this degassed-magma might have still been hot and slightly below its Solidus temperature (ca. 900 °C). Once emplaced and solidified, lava undergoes fracturing due to volume contraction and/or tectonics. Fractures are escape routes for the huge amounts of gas feeding the persistent plume^36^. In the shallowest portion of the volcano (the last 1300 m, in our instance), the most significant gas species are already exsolved (e.g. exsolution pressure of H_2_O ca. 250 MPa; SO_2_ ca. 140 MPa; CO_2_ > 400 MPa^37^), and cannot therefore modify the chemical behaviour of magma as in the case of gas flushing^38^. However, the gas are very hot and its continuous passage through the rock can lead to a notable heat release, which can trigger eruptions. The thermal contribution of the gas flux in originating the 28 December eruption can be tested starting from the observation that during the 134 days following the previous eruptive event (ended the 15^th^ of August, 2014), gas emitting, incandescent fractures (Fig. 2; Supplementary Fig. S3) were observed on the edges of SEC, proving that the gas, through its passage, was heating the lava plug underneath the summit craters. The heat carried by gasses represents the sum of the translational kinetic energy of its atoms and molecules and is therefore independent of the gas species, depending only from its absolute T; it can therefore be evaluated as:

1 $${{\rm{\Delta }}{\rm{\varepsilon }}}_{g}={\rm{nCvT}}$$

where n is moles number; Cv is specific heat at constant volume; T is the absolute temperature. One mole of gas at initial temperature of 1200 °C can supply an energy amount of 18412.5J. Considering that on those 134 days the cumulative amount of SO_2_ (Fig. 5) was 3.2 × 10^5^ tons and considering SO_2_/H_2_O = 0.1 and CO_2_/SO_2_ = 0.6 (in Aiuppa *et al*.^39^), adding 6.1 × 10^4^ tons of HCl (daily average from Gambino *et al*.^22^), and 8.4 × 10^3^ tons of HF (pers. comm. A. La Spina) in 134 days (from 15 August to 28 December 2014) then ~1.9 × 10^11^ moles of gas have passed through the plug and the summit craters, providing a ∆ε_g_ of 3.5 × 10^15^ J.

Due to the low conductivity of the basaltic lava (k~1 W/mK), the basalt in the plug remaining after the August eruption, even though solid, had to be still hot. A useful example of solid Etnean lava levees at 900 °C can be found in Barberi & Carapezza^40^ and Wright *et al*.^41^. Considering 1000 °C as a plausible temperature for melting onset, the volume of basalt (Wv), whose temperature could be raised by 100 °C and brought to melting by the heat supplied by the gas in 134 days (∆ε_g_), can be calculated as follows:

2 $${\rm{Wv}}={{\rm{\Delta }}{\rm{\varepsilon }}}_{g}/{\rm{\rho }}({\rm{L}}{f}+{\rm{c}}({\rm{Tm}}\mbox{--}{\rm{T}}1))$$

**Figure 7:**
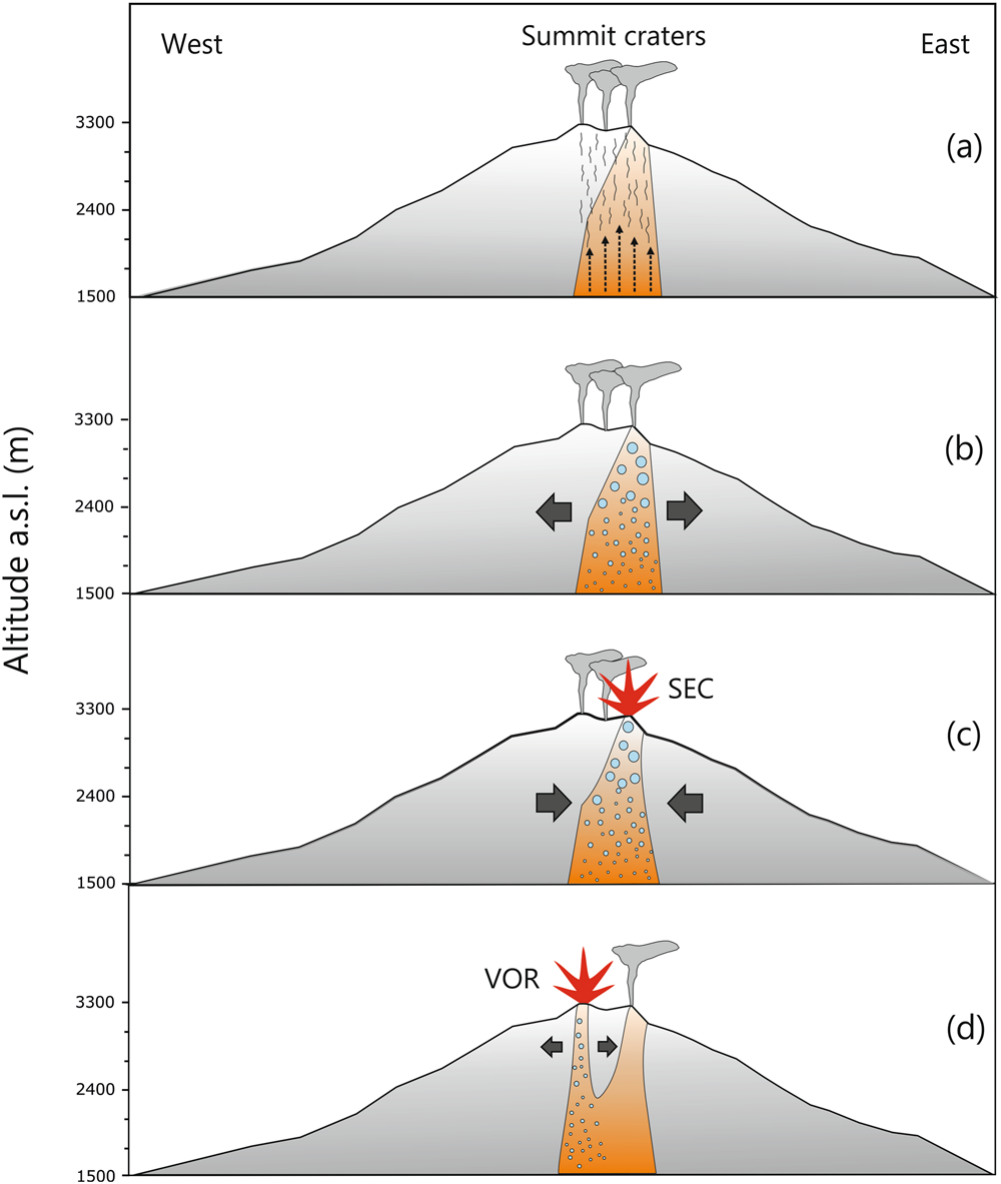
Sketch of the process leading to the 28 December 2014 eruptive event. (**a**) Pre-eruptive period from mid-August until the beginning of the event. Volcanic gas passes through the rigid fractured plug; (**b**) at 13:50 on 28 December (two and a half hours before the beginning of the event) the plastic threshold of the plug of magma is surmounted, vesiculation begins producing a positive strain acting on the upper portion of the edifice (cf. Fig. 6); (**c**) at 16:15 on 28 December the eruption begins, the discharge of the magma plug within the uppermost 1500 m of the edifice originates a negative strain (cf. Fig. 6), indicating a decompression until the end of the eruption (18:30); (**d**) the decompression caused by the magma discharge (evidenced by diverging arrows) triggers the ascent of deeper volatile-rich magma through the upper section of the VOR. This figure was created using CorelDRAW™ graphic suite software (http://www.corel.com/it/).

Where c = 1.4 × 10^3^ J/(kgK) is the specific heat of an Etnean basalt calculated using MELTS^42^, Tm ≈ 1000 °C is a lower limit for the onset of melting and Tl ~900 °C is a likely temperature of the basalt remaining in the plug. With a basalt density ρ = 2800 kg/m^3^ and a latent heat of L = 5.0 × 10^5^ J/kg (typical values for Etnean basaltic lavas^43–45^), melting fraction *f* = 0.7 (assuming a large partial melting of 70%), a magma volume of 2.5 million m^3^ is found. The lava emitted on 28 December was ~3.0 million m^3^, so most of the lava erupted could have been driven to melt by the heat carried by the gas flux. The estimate of ∆ε_g_ is fairly robust, against variations due to uncertainties, and demonstrates that the gas flux is thermally able to drive basalt melting. Indeed, the heat provided by the gas is released within a time span (134 days in the example), but the eruptive outburst is sudden and short lasting, as caused by a “threshold triggering”. The process could be envisaged as follows: the heating of the basalt in the viscous/rigid plug could have led to the softening at first and then to the very melting of the basalt (Fig. 7a). The melting needed for triggering the eruption was partial; we used a 70% estimate in the above thermodynamic calculation only to be conservative, but probably a lower degree of melting (50-30%) may have sufficed to trigger the eruption. The “critical” threshold was reached when the plug stopped behaving as a rigid body and became a plastic blob, less permeable than fractures on the rigid plug, to the upward migrating gasses (Fig. 7b). In fact, from 10 December the flux decreased, dropping to its minimum value of 440 t/d three days before the event. We might consider this lowermost value as the minimum permeability threshold. In this condition, in order to pass through the newly molten viscous basalt, the gas had to boil and produce bubbles. These bubbles generated, in the relatively short time of 2 hours, enough overpressure to break the resistance of the SEC plug and edifice and produce the violent outburst (Fig. 7c). We may speculate that the slight gas flux increment (~2000 t/d) observed on 26 and 27 December, might have supplied the critical energy to trigger the event.

Such a reconstruction of the eruption triggering explains well the data of the borehole strainmeters, showing a two-hour long contraction phase, corresponding to the overpressure build up due to the magma boiling; followed by the dilatational phase, associated to the eruptive discharge of the plug. Our reconstruction also accounts for the nature of the seven LP (long period) events from 16:11 and 16:50^22^ (Fig. 4a), which we relate to the overpressure build up in the plug. In fact, the timing and characteristics of these seismic events suggest that they were likely associated with a process of fluid-bubbles pressurization in a viscous magma. One exception is the seismic event recorded at 16:50 (local magnitude - Ml = 3.0), which can be classified as a “hybrid” (seismic events with a pronounced high frequency onset and a coda dominated by low frequency wave train, e.g. White *et al*.^46^). This could have been produced by rock volume failure at very shallow levels of the SEC edifice or by violent degassing into adjacent thermally induced cracks^47^. Furthermore, if to be erupted was the partially re-molten old degassed magma, the H_2_O content in the erupted lava (expressed as Loss On Ignition - L.O.I. Table 2) is expected to be low, as it is in the 3 samples taken on two lava flows. But also the lava must have been highly viscous and with remarkable yield strength, and in fact, the lava emitted has “aa” morphology, totally breached up in clinkers, very thin (~1 m thick or less) and almost lacking of the massive portion. Moreover, the entire edifice of SEC was broken apart from SW to NE, by a very peculiar eruptive fracture, which displayed huge niches similar to avalanche scallops on the NE and SW sides from 3050 m (a.s.l.) to the summit (see Fig. 1b,c; Supplementary Figs S1 and S2). These avalanche niches, together with the extremely high effusion rate >370 m^3^/s, indicate that on 28 December 2014 the ~3 million m^3^ of lava were not simply erupted but literally discharged from the SEC, similarly to what happens in andesitic exogenous lava domes.

**Table 2:** Major element (wt%)﻿ and minor ele﻿ments analyses of the lava erupted on 28 December 2014. CSE-DF and CSE-OF have been emitted by the south-western branch of the eruptive fracture, whereas the CSE-EST has been erupted by the north-eastern branch of the eruptive fracture.

Sample name	SiO_2_ (wt%)	TiO_2_	Al_2_O_3_	Fe_2_O_3_	MnO	MgO	CaO	Na_2_O	K_2_O
CSE-DF	48.03	1.93	16.85	11.24	0.18	4.12	11.84	3.21	2.1
CSE-OF	48.24	1.79	17.96	10.45	0.16	3.75	11.47	3.48	2.08
CSE-EST	47.96	1.85	17.48	10.89	0.17	4.06	11.86	3.28	2.04
	**P**_**2**_ **O**_**5**_	**L.O.I.(wt%)**	**Sr**	**V**	**Cr**	**Co**	**Ni**	**Zn**	**Rb**
CSE-DF	0.41	0.1	1074	422	40	137	18	89	40
CSE-OF	0.49	0.14	1066	418	46	135	17	81	39
CSE-EST	0.43	0	1071	413	38	140	16	86	39

Such a massive discharge, very rapid (~2 hours and 15 minutes), had probably induced a sudden decompression of the shallow plumbing system, thus triggering the ascent of rich-volatile magma from depth, which, exsolving at lower pressure, determined the observed syn-post eruptive dramatic deformation of the uppermost portion of the volcanic edifice (Supplementary Fig. S6b,c; Fig. 3b,d). Eventually, this magma fed the eruption at VOR, characterized by violent Strombolian activity (Supplementary Fig. S7) and by a sustained SO_2_ flux (Fig. 5).

The peculiarities observed in the unrest and on 28 December 2014 at Mount Etna are somewhat dissimilar to typical basaltic volcanoes behaviour. The geophysical, gas geochemistry and field observations found a balanced explanation if we interpret the eruption as the discharge of a re-melted basalt plug together with the uppermost portion of the SEC edifice, rather than the classic eruptive dynamics fed by fresh magma rising from depth. In this reconstruction, the role of the volatiles extraneous to the erupting magma and coming from below is determinant in triggering the eruption^38^. The proposed eruptive mechanism envisages a scenario that is similar to what commonly observed on evolving exogenous domes usually associated with andesitic or more acidic magmas. Obviously, the SEC cannot be defined as a “dome”, neither can the event described here be ascribed to a typical felsic dome collapsing. This process, which lacks a proper terminology, represents a novel eruptive mechanism at Mount Etna. Whether this mechanism is unique or indeed could be more common than expected at basaltic volcanoes, is an open issue for further studies. There have been other eruptive events at the SEC characterized by high discharge rates in limited time^18, 19^, in the light of our considerations, the eruptive mechanisms behind these events might be revisited. We believe that this work represents a significant step forward in understanding eruptive unrest at Mount Etna and it might be an example to re-evaluate eruptive unrest at basaltic volcanoes worldwide.

## Methods

### Deformation and modelling of volcanic sources

The Continuous GPS (CGPS) network of Mt. Etna volcano, managed by the Istituto Nazionale di Geofisica e Vulcanologia, Osservatorio Etneo (INGV-OE), consists of 38 stations located on the summit area and flanks of the volcanic edifice. Raw data collected between August 2014 and March 2015 have been processed using the GAMIT/GLOBK software packages^48, 49^, applying the method discussed in Bruno *et al*.^50^. The deformation patterns were also interpreted with analytical time-invariant models. In order to estimate spatial coordinates and geometrical parameters of the magmatic source in the pre-eruptive phase, we used the Yang *et al*.^51^ model, while we modelled a rectangular tensile dislocation^52^ for the intrusion of the evening of December 28. Both models take into account the influence of topography on the final deformation pattern by applying the method proposed in Williams & Wadge^53^. The applied procedure is described in Bruno *et al*.^54^.

### Petrochemical analysis

Whole-rock analyses were carried out at the laboratories of the Department of Physics and Earth Science of the University of Ferrara (Italy) on powdered aliquots of rocks. Whole-rock major concentrations were measured by X-ray fluorescence (Thermo ARL Advant XP). Intensities were corrected for matrix effects using the method of Lachance & Traill^55^. Loss on ignition (L.O.I.) was determined by gravimetric method assuming Fe_2_O_3_ as 15% FeO. Accuracy and precision are better than 2–5% for all major elements (Table 2).

### SO_2_ flux

The daylight bulk sulphur dioxide (SO_2_) flux from the summit craters of Mt. Etna was measured by the FLAME-Etna (FLux Automatic MEasurements) network. The network consists of ten ultraviolet scanning spectrometer stations spaced ~7 km apart and installed at an altitude of ~900 m a.s.l. on the flanks of Mt. Etna^56, 57^. Each station scans the sky for almost 9 h, intersecting the plume at a mean distance of ~14 km from the summit craters and acquires a complete plume-scan in ~5 min. Open-path ultraviolet spectra are reduced on site applying the DOAS technique and using a modelled clear-sky spectrum^57, 58^. Inverted data are in real-time transmitted to the INGV, Osservatorio Etneo, where SO_2_ emission rates are computed; uncertainty in flux range between –22 and +36%^59, 60^. A detailed description of plume SO_2_ flux measurement method can be found in Calvari, *et al*.^56^, Salerno *et al*.^59^.

## Electronic supplementary material

Supplementary Information

Supplementary information:video
