# Supplementary material for: Dome-like behaviour at Mt. Etna: The case of the 28 December 2014 South East Crater paroxysm
Source: Sci Rep. 2017 Jul 13;7:5361. doi: 10.1038/s41598-017-05318-9 (PMC5509668; doi:10.1038/s41598-017-05318-9)
Supplement: Supplementary file 1 — Supplementary Information [file 41598_2017_5318_MOESM1_ESM.pdf]

Submitted to Scientific Reports

Original Article

Dome-like behaviour at Mt. Etna: The case of the 28 December 2014 South East Crater paroxysm

Ferlito, C.[1], Bruno V.[2], Salerno G. [2] (*), Caltabiano T.[2], Scandura D.[2], Mattia M.[2] and Coltorti M.[3]

[1]Università degli Studi di Catania, Dipartimento di Scienze Biologiche, Geologiche ed Ambientali, Corso Italia 57, Catania (Italy)

[2]Istituto Nazionale di Geofisica e Vulcanologia, Osservatorio Etneo, Piazza Roma 2, Catania (Italy)

[3]Università degli Studi di Ferrara, Dipartimento di Fisica e Scienze della Terra, Via Saragat 1, Ferrara (Italy)

(*) Corresponding author: Dr. Giuseppe Salerno, e-mail address: giuseppe.salerno@ingv.it

Supplementary Material

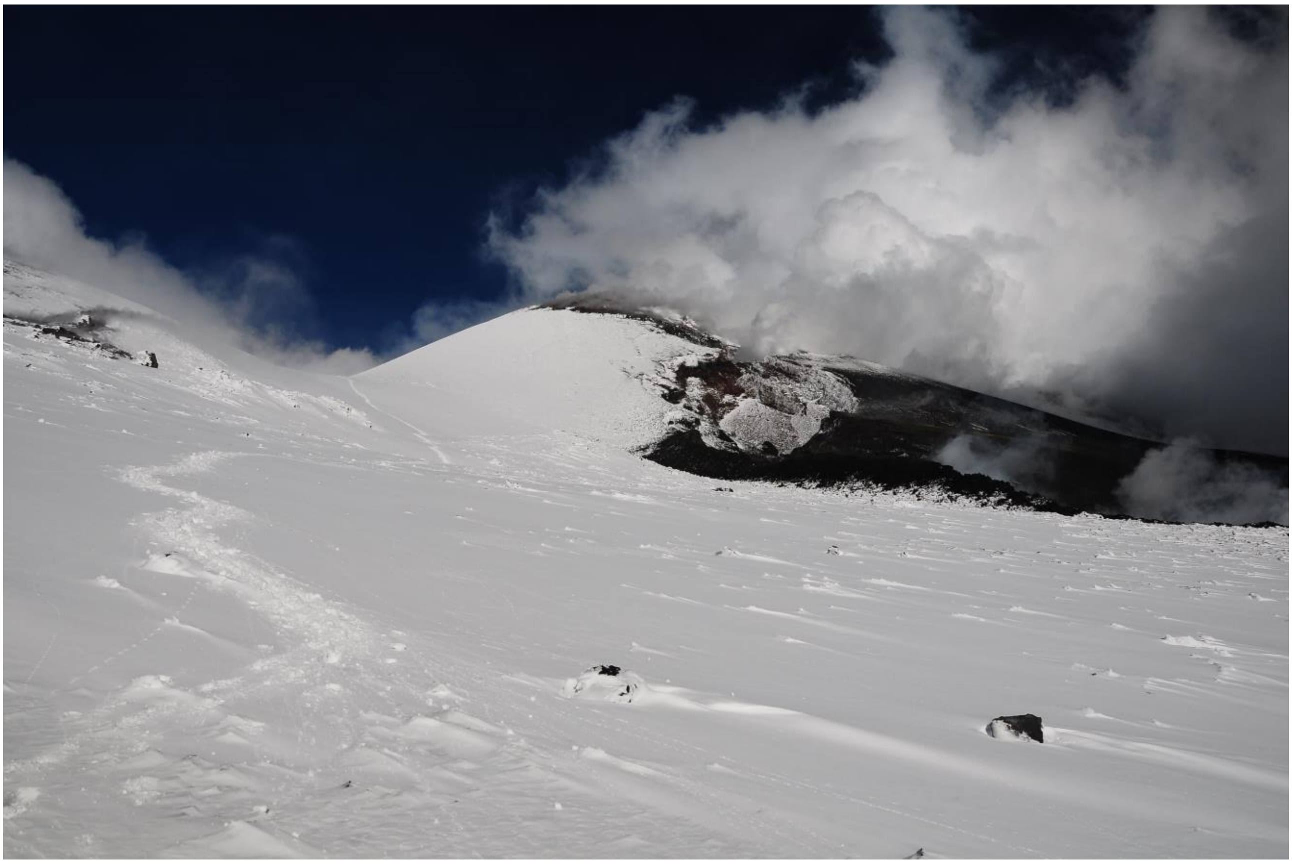

Supplementary Fig. S1. The large “avalanche” scallop representing the tip of the south-western branch of the eruptive fracture (3050 m a.s.l.), from this view angle (southwest) it can be seen that the entire cone of SEC was affected by this feature. Photo taken by Ferlito C.

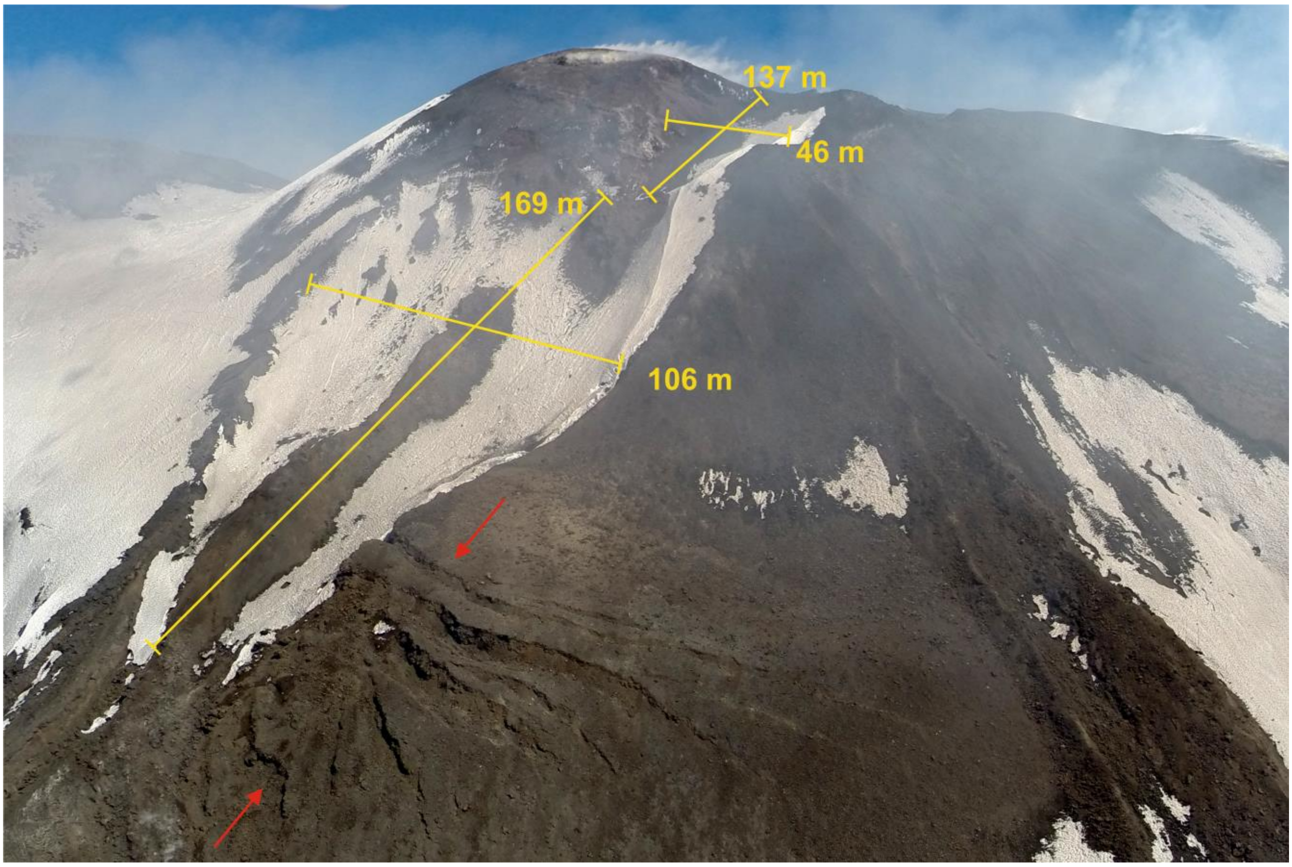

Supplementary Fig. S2. Photo of the scallop on the southwest flank of the SEC. The syn-eruptive fractures (between red arrows) are visible to the right of the scallop. The photo was taken with a drone overflight on 16 April 2015 and georeferenced; in this way we were able to give measurements of the scallops. Photo taken by Antonio Zimbone (ETNA WALK).

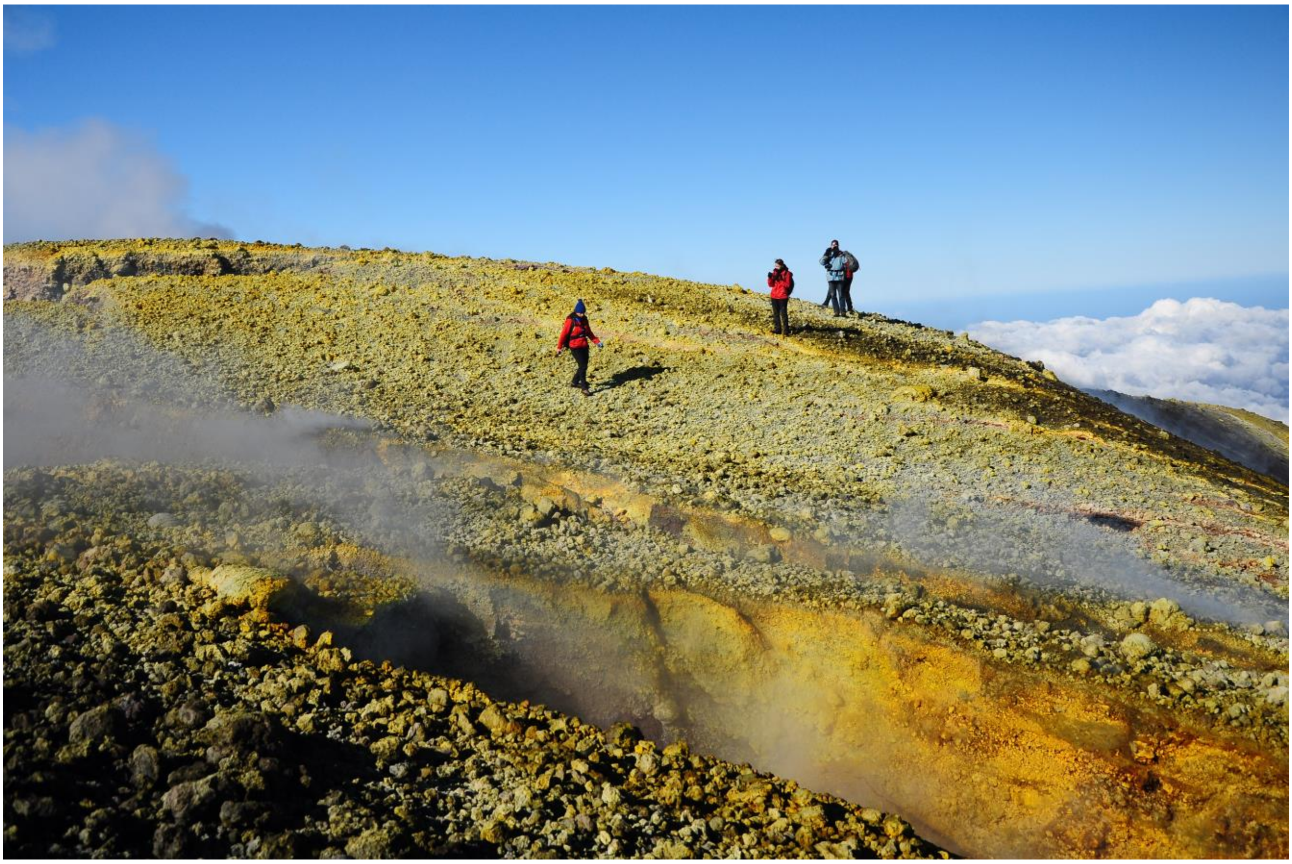

Supplementary Fig. S3. Large fracture (about 2 m wide) on the northern edge of the SEC summit. The photo was taken on 1 December 2014. Photo taken by Ferlito C.

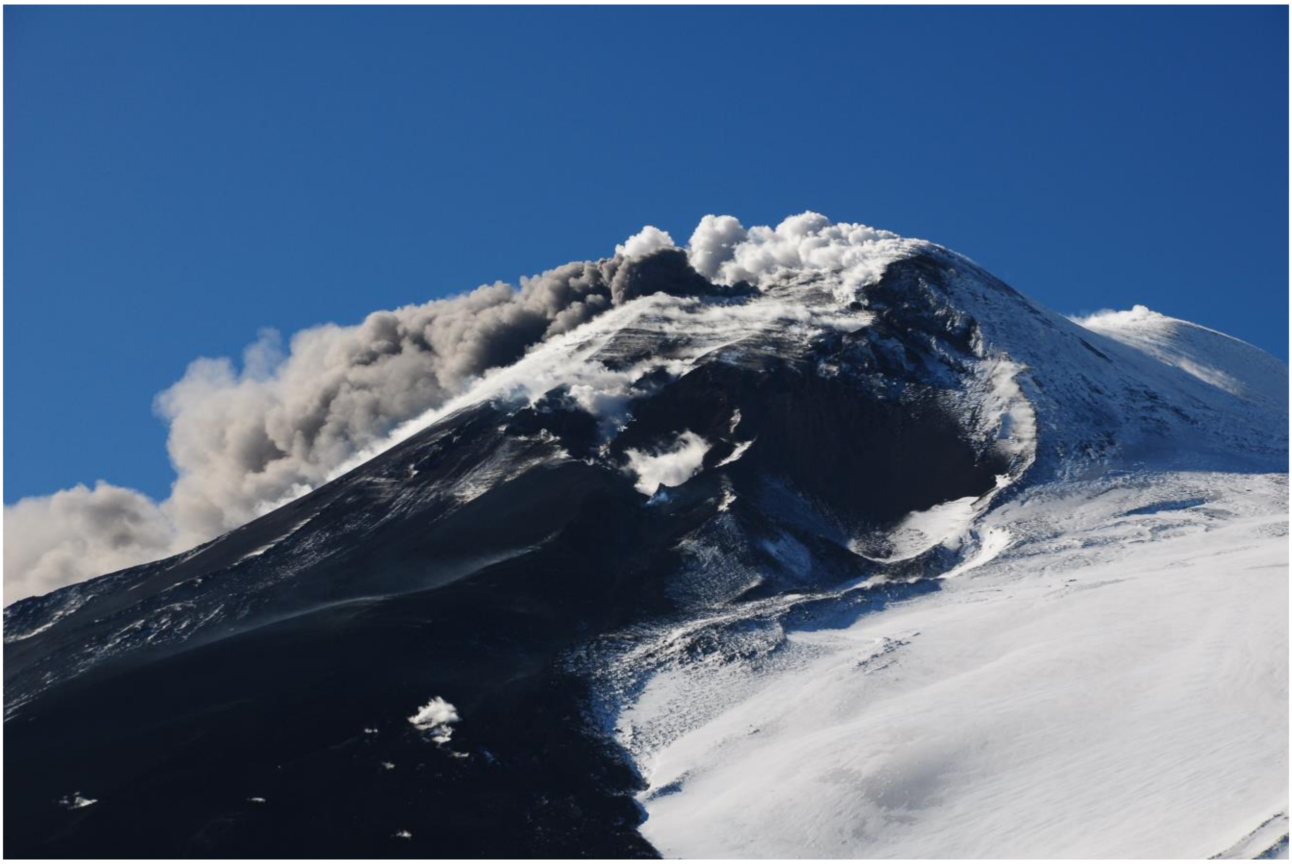

Supplementary Fig. S4. The northeastern face of the SEC with the ash cloud emission from its summit after the 28 December 2014 event. The photo was taken on 2 January 2015 from the northern ridge of the Valle del Bove. Photo taken by Ferlito C.

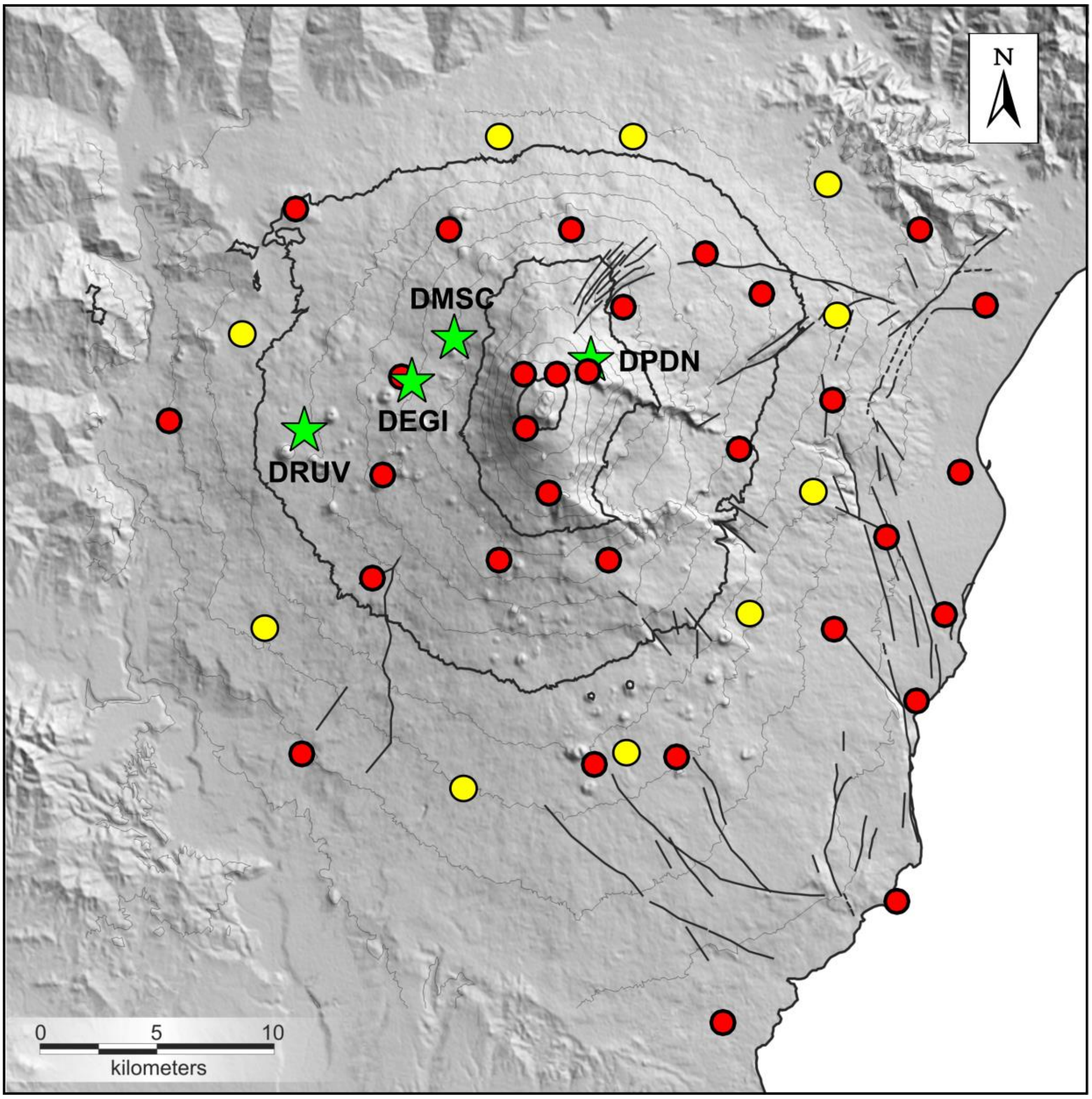

Supplementary Fig. S5. Map of the instrumental networks: the red circles are the CGPS stations; the yellow circles the stations of the FLAME network; the green stars are the strainmeters.

The topography is based on a DEM held by INGV-OE, Cartography Lab (http://geodb.ct.ingv.it:8088/geonetwork/srv/ita/main.home).

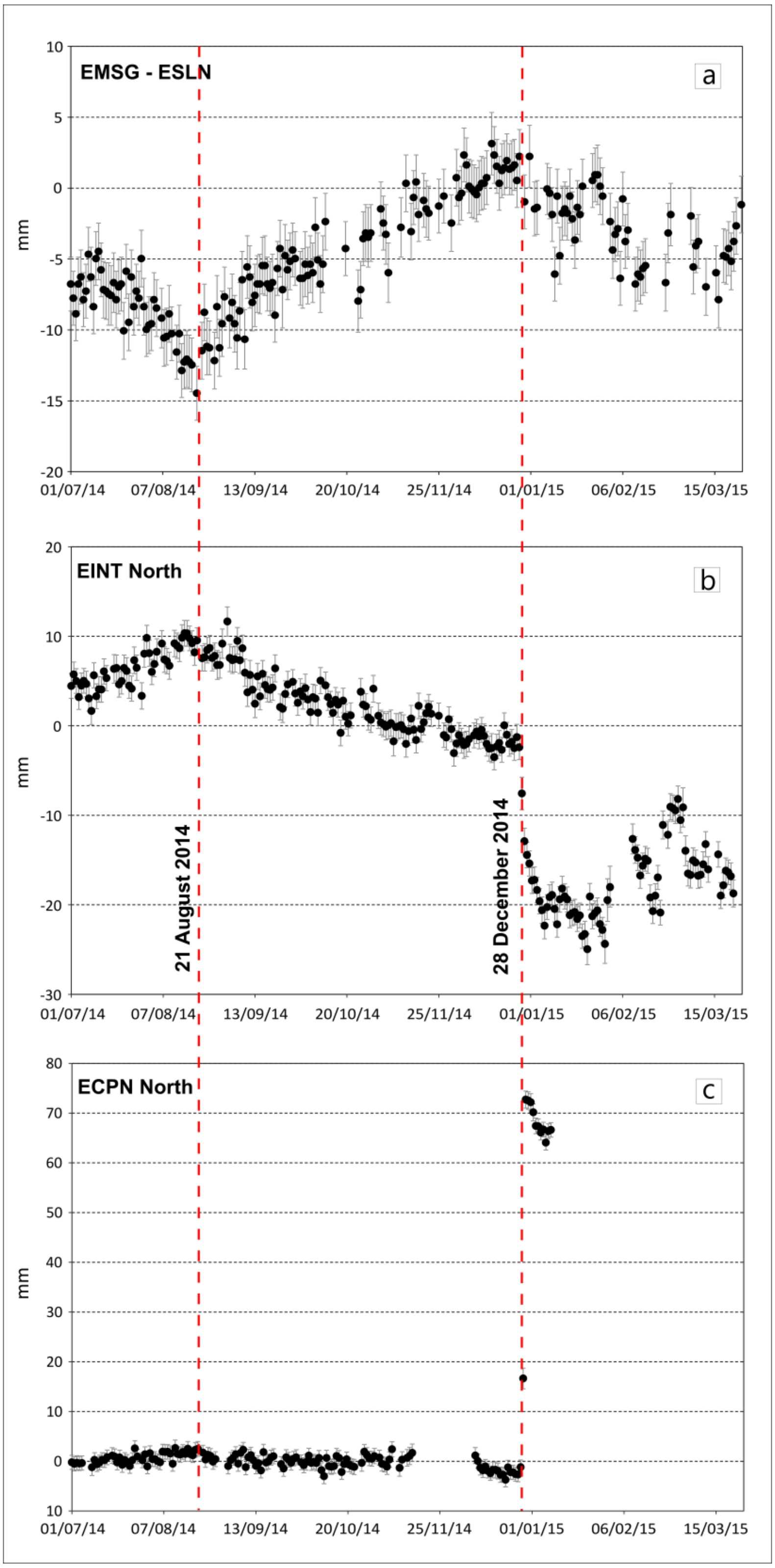

Supplementary Fig. S6. a) Time series of the length variations between the EMSG and ESLN CGPS stations. Vertical dotted lines separate phases characterized by different deformation behaviour, recognized on the basis of changes in the slope of the baseline, or sudden variations. b, c) North component of the position time series of the EINT and ECPN CGPS stations. The red dashed line indicates the onset of the 28 December 2014 paroxysm.

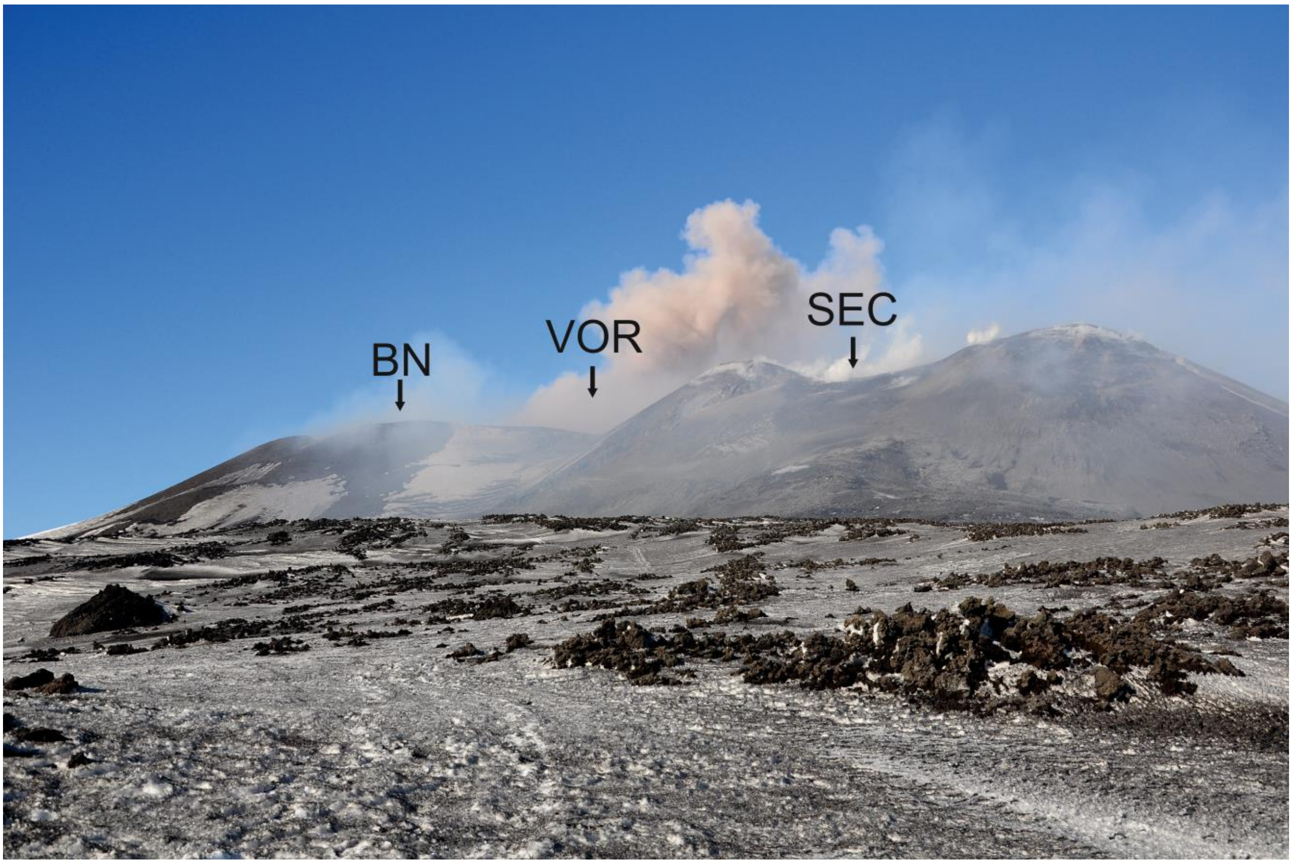

Supplementary Fig. S7. View of the Etnean summit craters from south-southeast. The brown cloud was produced/generated by the strombolian activity at the VOR. The photo was taken on 8 January 2015. Photo taken by Ferlito C.
